# Adaptive evolution of chloroplast genome structure inferred using a parametric bootstrap approach

**DOI:** 10.1186/1471-2148-6-13

**Published:** 2006-02-09

**Authors:** Liying Cui, Jim Leebens-Mack, Li-San Wang, Jijun Tang, Linda Rymarquis, David B Stern, Claude W dePamphilis

**Affiliations:** 1 Department of Biology, Institute of Molecular Evolutionary Genetics, and Huck Institutes of Life Sciences, The Pennsylvania State University, University Park, PA 16802, USA; 2 Department of Biology, University of Pennsylvania, Philadelphia, PA 19104, USA; 3 Department of Computer Science and Engineering, University of South Carolina, Columbia, SC 29208, USA; 4 Boyce Thompson Institute, Cornell University, Ithaca, NY 14853, USA

## Abstract

**Background:**

Genome rearrangements influence gene order and configuration of gene clusters in all genomes. Most land plant chloroplast DNAs (cpDNAs) share a highly conserved gene content and with notable exceptions, a largely co-linear gene order. Conserved gene orders may reflect a slow intrinsic rate of neutral chromosomal rearrangements, or selective constraint. It is unknown to what extent observed changes in gene order are random or adaptive. We investigate the influence of natural selection on gene order in association with increased rate of chromosomal rearrangement. We use a novel parametric bootstrap approach to test if directional selection is responsible for the clustering of functionally related genes observed in the highly rearranged chloroplast genome of the unicellular green alga *Chlamydomonas reinhardtii*, relative to ancestral chloroplast genomes.

**Results:**

Ancestral gene orders were inferred and then subjected to simulated rearrangement events under the random breakage model with varying ratios of inversions and transpositions. We found that adjacent chloroplast genes in *C. reinhardtii *were located on the same strand much more frequently than in simulated genomes that were generated under a random rearrangement processes (increased sidedness; p < 0.0001). In addition, functionally related genes were found to be more clustered than those evolved under random rearrangements (p < 0.0001). We report evidence of co-transcription of neighboring genes, which may be responsible for the observed gene clusters in *C. reinhardtii *cpDNA.

**Conclusion:**

Simulations and experimental evidence suggest that both selective maintenance and directional selection for gene clusters are determinants of chloroplast gene order.

## Background

The influence of genotype on phenotype is not limited to the coding of peptides and functional RNAs by nucleotide sequences. An organism's phenotype is also affected by the chromosomal arrangement of genes and the interaction of gene products. Comparative genomics has revealed a variety of gene clusters and chromosomal segments that have remained intact over hundreds of millions of years [1]. Selection for clustering of co-transcribed genes has been hypothesized to influence gene order within bacterial and organelle genomes, where gene clusters typically encode multiple components of a functional pathway [2]. For example, the ribosomal proteins are encoded by similar operons in archaebacteria, eubacteria and plastids [3]. In eukaryotic genomes, co-expression of neighboring genes is significantly associated with the functional roles of the genes (such as housekeeping genes or genes in the same metabolic pathway) [4,5]. One way that those genes become clustered is through tandem duplication, which usually results in functionally related genes being adjacent. On the other hand, unrelated genes may also be brought together through chromosome rearrangements (recombination, inversion and transposition).

Unless selection is acting to maintain or promote gene clusters, gene orders in genomes subjected to rearrangements should become randomized with respect to function or co-expression profiles. Significant clustering has been inferred using permutation tests that compare observed physical distances between pairs or blocks of co-expressed or functionally related genes to a null distribution constructed from randomized gene orders [4,5]. However, this approach is limited since the evolutionary history of the genome was not considered. When comparing gene orders among related species, it is possible to estimate the ancestral genome and to simulate a null distribution for changes in gene order using a model. This evolutionary approach can be used to test directly the influence of selection on genome structure, that is, whether present-day genome structure has been influenced by directional selection for clustering of functionally related genes.

Small genomes, especially those of organelles and bacteria, are well suited to global comparisons of gene order. Like eukaryotic genomes, they are subject to structural changes such as inversion, transposition or translocation, as well as gene loss and (more rarely) gene gain. Chloroplast DNAs in most land plants share a highly conserved gene content and similar gene orders [6]. Most cpDNAs include two identical regions in opposite orientations called the inverted repeat (IR), flanked by large single copy (LSC) and small single copy (SSC) regions. The IRs generally contain the bacterial-like rRNA gene clusters, and the genes involved in photosynthesis (photosystem I/II, cytochrome b_6_/f, and ATP synthase) are arranged similarly in chloroplast and cyanobacterial genomes [2,3,7]. Despite these well-characterized patterns, it is unknown to what extent the conserved gene order reflects a slow intrinsic rate of neutral chromosomal rearrangements, rather than selection against alternative gene orders. A model of neutral rearrangement of gene order is required to test formally whether gene orders evolve under selection which prefers some gene arrangements over others.

Nadeau and Taylor first proposed a model for the neutral evolution of gene order in comparisons of mouse and human chromosomes [8]. This "random breakage model" provides a null hypothesis for the evolution of gene order. It assumes a random distribution of break points and allows all possible gene orders without restrictions. The random breakage model has been used to infer organismal phylogenies from gene order data [9]. The gene order difference can be measured using the inversion distance, which is the minimal number of inversions necessary to transform one gene order to another. Currently, the most accurate heuristic approach is implemented in the GRAPPA software [10], which is generally suitable for small taxon sets because the algorithm scores inversion medians for all nodes iteratively across all possible phylogenies. Algorithms for genomes with arbitrary rearrangements, a few deletions and duplications have been developed [11], and the capacity of GRAPPA can be scaled up with the discovering method (DCM) to potentially very large data sets [12].

The random breakage model does not account for recombination hotspots, which have been reported from human-mouse genome comparisons [13]. However, at this time it may be difficult to model these hotspots, because the precise locations of reused breakpoints are unknown due to insufficient resolution of gene orders and potential errors in homology assessment given the scale of eukaryotic chromosomes [14]. Thus, the fragile breakage model [13], as an alternative to the random breakage model, has not been well established.

Whereas gene order is generally conserved among land plant cpDNAs, very little synteny is observed between this group and cpDNAs of the chlorophytic green algae *C. reinhardtii *[15,16] and *Chlorella vulgaris *[17]. The apparently increased rearrangement rate is associated with invasion by a large number of short dispersed repeats (SDRs), for which the evolutionary distribution is still poorly defined. The large number of rearrangements provides an excellent opportunity to test whether natural selection has preferred some changes in gene order. Here we present novel statistics and parametric tests that lead us to reject the models of random rearrangement in favor of directional selection for clustering of functionally related genes in *C. reinhardtii*. We also present experimental evidence that adaptive evolution of chloroplast genome structure could be driven by the advantage of concerted regulation conferred by polycistronic transcription.

## Results

### Functional clusters are not randomly distributed

**Figure 1:**
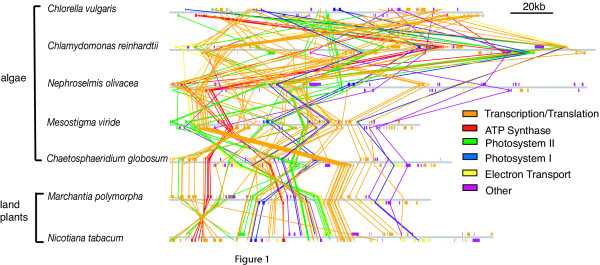
**Extensive rearrangement in *Chlamydomonas reinhardtii *and *Chlorella vulgaris *cpDNAs**. Representative cpDNAs from land plants and green algae are arranged to reflect their phylogenetic relationships. The scale bar indicates 20 kb. Each genome is linearized and drawn as a grey bar. Genes are drawn as colored rectangles and with those encoded on the positive strand above the genome bar. Colored lines connect the homologs included in this study and the functional category is shown by specific colors.

We compared gene orders of representative cpDNAs from land plants, including tobacco (*Nicotiana tabacum*, [GenBank:NC_001879]) [18] and liverwort (*Marchantia polymorpha*, [GenBank:NC_001319]) [19], a charophytic green alga (*Chaetosphaeridium globosum *[GenBank:NC_004115]) [20], chlorophytic green algae (*Nephroselmis olivacea *[GenBank:NC_000927] [21], *C. vulgaris *[GenBank:NC_001865] [17], *C. reinhardtii *[GenBank:BK000554] [16]), a green flagellate alga with uncertain affinities (*Mesostigma viride *[GenBank:NC_002186]) [22], and the plastid of *Cyanophora paradoxa *[GenBank:NC_001675] [23] (Figure 1) (Additional file 1, Additional file 3).

To measure the genome structure in terms of clustering by chromosome locations and gene functions, we defined "sided blocks" as contiguous genes coded on the same strand of the plastid chromosome, and "functional clusters" as blocks of functionally related genes (see Methods). The randomness in the observed distribution of shared genes in chloroplast genomes with respect to gene function was assessed using a Kolmogorov-Smirnov test. The null hypothesis was rejected in all seven cpDNAs investigated for genes in functional categories such as ATP synthases and electron transport (p << 0.05, Table 1). While this test suggests some degree of functional clustering in all chloroplast genomes, it does not take into account the phylogenetic relationship of these organisms, so it is unclear whether functional clustering in chloroplast genomes is a legacy of genome organization in a cyanobacteria-like ancestor, or the product of selection on gene order in the face of genome rearrangements.

**Table 1:** The Kolmogorov-Smirnov test of gene clustering by the functional category in cpDNAs §

cpDNA	*D*_*n *_(p-value)			
	Translation and transcription	Photosystem I and II	Electron Transport	ATP synthase
*Chlorella*	0.214(.6418)	0.488(.0066)	0.750(.0000)	0.833(.0000)
*Chlamydomonas*	0.198(.6866)	0.473(.0060)	0.780(.0000)	0.769(.0000)
*Nephroselmis*	0.209(.6207)	0.484(.0046)	0.703(.0000)	0.846(.0000)
*Mesostigma*	0.275(.2786)	0.549(.0008)	0.769(.0000)	0.846(.0000)
*Chaetosphaeridium*	0.242(.4388)	0.484(.0046)	0.714(.0000)	0.846(.0000)
*Marchantia*	0.341(.0986)	0.473(.0060)	0.714(.0000)	0.846(.0000)
*Nicotiana*	0.264(.3283)	0.473(.0060)	0.769(.0000)	0.846(.0000)

§The test statistic *D*_*n *_measures whether the distribution of functionally related genes is random in gene clusters. Total 85 shared genes between seven cpDNAs were included.

### Extensive rearrangements from the ancestral chloroplast genome to *C. reinhardtii*

**Figure 2:**
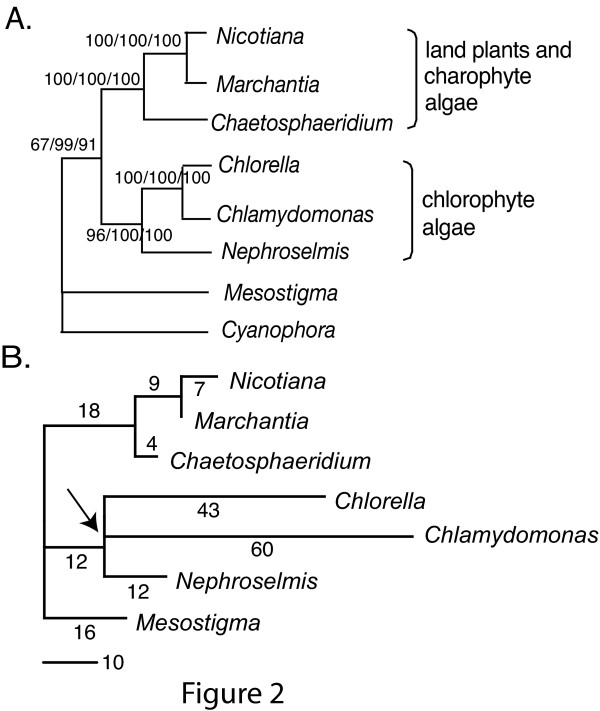
**The phylogeny of cpDNAs**. (A) The cpDNA phylogeny based on analysis of 50 concatenated proteins. The phylogeny includes major green plant and algal lineages and the outgroup *Cyanophora*. The bootstrap support values from maximum parsimony/neighbor joining/maximum likelihood analyses are labeled near each node. (B) Estimated inversion distances considering 85 common genes on the cpDNA phylogeny. There is an increase of rearrangements on branches leading to *C. reinhardtii*. and *C. vulgaris*, from a common ancestor indicated by an arrow.

In order to investigate evolutionary changes of gene order, we constructed a phylogeny of seven representative cpDNAs and rooted with the sequence of *C. paradoxa *[23]. Maximum parsimony, neighbor joining and maximum likelihood analyses of an alignment of 50 concatenated protein sequences including a total of 19,836 aligned sites (Additional file 2), all yielded identical fully resolved topologies with high bootstrap support (Figure 2A). *Mesostigma *was placed as a basal charophyte lineage in one previous analysis [24]. The unrooted phylogeny of seven cpDNAs (Figure 2B) is congruent with the alternative placement of *Mesostigma *either as a basal charophyte [24] or basal to both charophyte and chlorophyte lineages [22]. This tree was used as the reference phylogeny for gene order inference.

We scored the orders of 85 genes shared in the seven genomes (Gene orders are in additional file 3). Then we used modified versions of GRAPPA [11,25] to compute the inversion distance between ancestral nodes and each terminal node (Figure 2B; see Methods). The branches leading to two chlorophytic green algae, *C. reinhardtii *and *C. vulgaris*, are much longer than the branches leading to the other taxa, and that many more steps were inferred on the *C. reinhardtii *lineage relative to the *C. vulgaris *lineage. Gene duplications or deletions were mapped before scoring the ancestral genomes with inversions, and were not counted as rearrangements. IRs were present in all inferred ancestral nodes, and one copy was lost in *C. vulgaris*. Ancestral gene orders were scored on all the phylogenies using a two-step approach (see Methods). Due to the computational time limit (the full search for ancestral gene orders may require months), we stopped scoring all possible ancestral gene orders with the data set after 25 days and took the best scored ancestral gene orders at that time (Additional file 4).

The cpDNAs of two land plants, *N. tabacum *and *M. polymorpha*, were separated by an estimated 7 inversions based on the data set. One large inversion (~30 kb) in the LSC region has long been recognized to separate the two genomes [26]. Additional rearrangements are directly observable through comparison of gene order files for the two species (see additional file 5 for the sequences of gene order rearrangements). Using GRAPPA, all rearrangements were inferred as inversions, but the total number of inversion events estimated by GRAPPA may be greater than the true (but unknown) mixture of inversions and transpositions because one transposition could result in the same change in gene order as two or three inversions.

### Increased order in the genome structure after rearrangements

**Figure 3:**
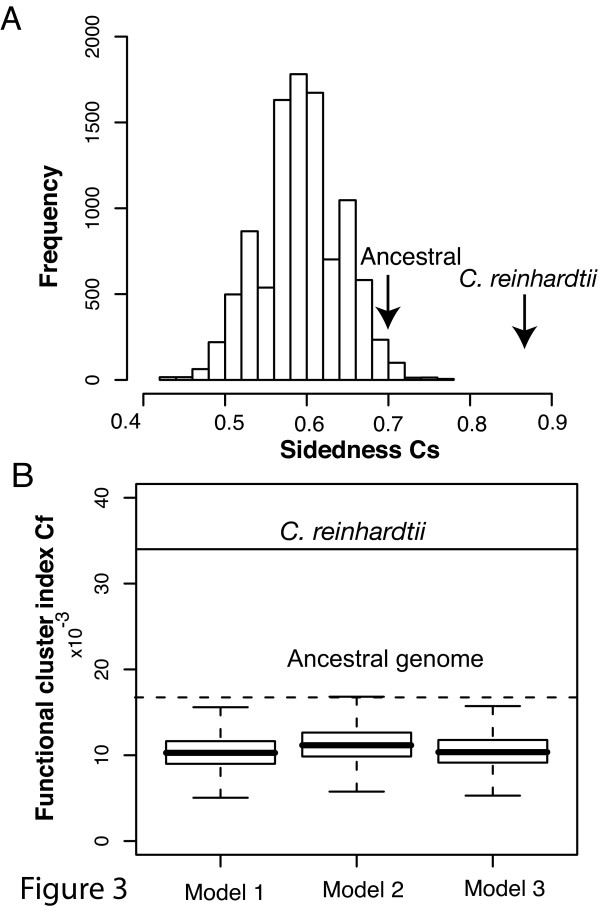
**Comparison of sidedness and functional cluster indices in *C. reinhardtii *cpDNA to those of simulated genomes**. (A)The sidedness index C_s _observed in *C. reinhardtii *(indicated by an arrow) is significantly larger than C_s _of gene orders simulated under the random breakage model (inversion only) and the estimated ancestral genome indicated in Figure 2B. (B) The functional cluster index C_f _for *C. reinhardtii *(indicated by a solid horizontal line) is greater than that for the inferred ancestral genome (dashed line), in contrast to the decrease predicted by three sets of simulations under the random breakage model. Models 1, 2 and 3 specified the inversion/transposition ratios to be 1:0, 10:1 and 1:1, respectively, in simulations with 10,000 replicates. The box section of the box plot indicates the first quartile, median and the third quartile of the distribution.

Two genomic structural characteristics were measured: the propensity of adjacent genes to be clustered on the same strand (using the sidedness index C_s_) and the clustering of functionally related genes (using the functional cluster index, C_f_) (see Methods). Both indices were calculated for the inferred ancestral gene orders and extant daughter lineages. Among land plants and charophytes, the inferred sidedness among ancestral genomes was similar to extant lineages, however, among the chlorophytes an opposite trend was observed, especially in the *C. reinhardtii *lineage (Additional file 3). The large number of rearrangements in the *C. reinhardtii *cpDNA lineage resulted in dramatically increased sidedness relative to the inferred most recent common ancestor of *C. reinhardtii *and *C. vulgaris *(C_s _ancestor = 0.6966, C_s _observed = 0.8710; Figure 3A). A small increase of C_s _was found in the *N. olivacea *lineage and there was almost no change in the lineage leading to *C. vulgaris*. A large increase was also observed in the functional clustering index, C_f_, for *C. reinhardti *(C_f _ancestor = 0.01674, C_f _observed = 0.03397; Figure 3B), whereas the trend was less profound in other lineages (Additional file 3). Thus, even if the ancestral genome already had a "sided" structure, sidedness increased with genome rearrangements as *C. reinhardtii *cpDNA evolved. The inferred increase in sidedness and functional clustering in the face of the large number of rearrangements on the lineage leading to *C. reinhardtii *might be adaptive, if such increases were not expected under random rearrangements.

To test the null hypothesis that the changes in C_s _and C_f _were consequence of random genome rearrangements rather than a consequence of directional selection (H_0_: random rearrangement; H_A_: constraints in rearrangements), we simulated random rearrangements starting with the inferred ancestral genome along the branch leading to *C. reinhardtii*. Although inversions are the most abundant type of rearrangement in cpDNAs [27], we also considered the contribution of transpositions under three inversion to transposition ratios, while the total number of rearrangements was fixed according to the branch length inferred using GRAPPA (Figure 2B). Three simulations with 10,000 replicates were conducted with inversion to transposition ratios of 1:0, 10:1 and 1:1 under the random breakage model. The mean C_s _values for the three sets were 0.5929, 0.6084 and 0.5948, respectively, and the 95% confidence intervals were (0.5056,0.6742), (0.5281, 0.6854) and (0.5169, 0.6742), respectively. All datasets simulated under the random breakage model showed a significant decrease of sidedness from the ancestral level (p < 0.0001). In contrast, the C_s _value calculated for *C. reinhardtii *increased significantly to 0.8710 (Figure 3A), greatly exceeding the sidedness that would be expected in a genome that had undergone this much evolutionary change relative to its ancestor. Simulations using inferred ancestral genomes for land plant lineages (e.g. *N. tabacum*) also strongly reject the null hypothesis of random rearrangements (results not shown).

Given the large number of rearrangements observed in the *C. reinhardtii *lineage, C_f _was also predicted to decrease significantly under the random breakage model, but C_f _did not decrease as observed in *C. reinhardtii *(Figure 3B). The simulations with three models described above (all inversions, a small fraction of transpositions, and equal inversions and transpositions) all yielded a large decrease in clustering as expected (the observed C_f _in *C. reinhardtii *is 0.03397, and the 95% confidence intervals for C_f _in simulated genomes were 0.00744–0.01401, 0.00812–0.014299 and 0.0750-0.01418, respectively). When transposition was included in simulations, decreases of C_f _were on a similar scale to the inversion-only simulations. Taken together, these results indicate that the remarkable increase in sidedness and functional clustering observed in *C. reinhardtii *cpDNA has not been the outcome of solely chance events. Instead, the strong deviation from the range of outcomes expected under various random breakage models implies that the genome structure is the outcome of a directional selective process.

**Figure 4:**
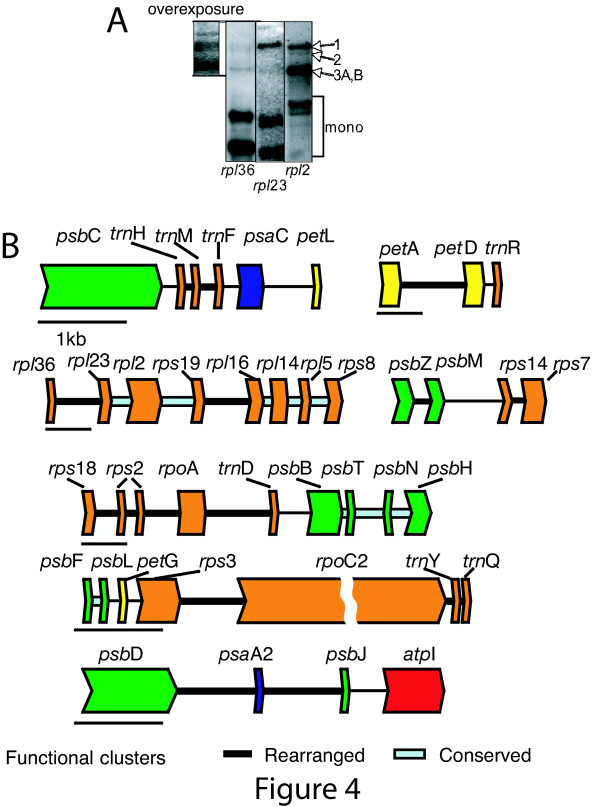
**Selected functional clusters from *C. reinhardtii *cpDNA**. (A) Evidence for co-transcription of the genes *rpl*36-*rpl*23-*rpl*2-*rps*19. The gel was loaded with total RNA from wild-type cells, and shows new evidence for co-transcription (see text). The top left lane is an over-exposure of the *rpl*36 gel. Transcripts 1 and 2 (3.5 and 3.3 kb) are tricistronic *rpl*36-*rpl*23-*rpl*2, transcript 3A (2.5 kb) is *rpl*36-*rpl*23, and transcript 3B (2.5 kb) is probably *rpl*2-*rps*19. Single gene transcripts are labeled "mono". (B) Rearranged functional clusters, which were absent from the inferred common ancestor of *C. reinhardtii *and *C. vulgaris*, were identified in *C. reinhardtii *(genes connected by bold black lines). Cyan lines connect conserved clusters retained from the ancestor cpDNA. The genes are displayed in the coding direction, and from top to bottom relative to their order in the genome. The exception is *psb*N, which is on the opposite strand relative to other genes shown (*psb*T-B-N-H). A scale bar of 1 kb is shown below and at the left of each gene cluster.

The increased level of organization in *C. reinhardtii *cpDNA was associated with both maintenance of ancestral clusters and growth of new clusters. There were six conserved blocks containing 19 of the 85 genes shared between the *C. reinhardtii *and the *C. vulgaris *cpDNAs. These blocks include concentrations of genes from a single functional category, such as ribosomal proteins (*rpl*23-*rpl*12-*rps*19, *rpl*16-*rpl*14-*rps*8), Photosystem II (*psb*L-*psb*F, *psb*B-*psb*T-*psb*N-*psb*H), translation apparatus (*rrn*16-*trn*I-GAU – *trn*A-UGC – *rrn*23-*rrn*5), and ATP synthase subunits (*atp*F-*atp*H). Moreover, a number of small clusters of functionally related genes inferred in the ancestral genome were brought together in *C. reinhardtii *("rearranged clusters" in Figure 4B). These include transcription/translation genes (*trn*H-M-F; *rpl*/*rps*; *rps*3-*rpo*C2), electron transport genes (*pet*A-*pet*D), and photosynthetic genes (*psb*D-*psa*A exon 2-*psb*J) (Figure 4B). The new clusters contributed to the increase of C_f _in the *C. reinhardtii *chloroplast genome.

### Coordinated expression of genes in functional clusters

Co-transcription of several clusters shown in Figure 4B has been previously documented, including *psb*D-*psa*A exon 2-*psb*J-*atp*I [28], *psb*F-*psb*L [29], *pet*A-*pet*D [30], and *psb*M-*psb*Z [31]. Co-transcription of *rpl *and *rps *genes has been found in land plant chloroplasts [32]. We documented co-transcription for an additional novel functional cluster, shown in Figure 4A. Using RNA gel blots, tricistronic transcripts of *rpl*36-*rpl*23-*rpl*2 and possibly dicistronic *rpl*2-*rps*19 species could be detected. Taken together, it appears that the clusters of functionally related genes observed in *C. reinhardtii *cpDNA may be frequently co-transcribed.

## Discussion

By reconstructing the possible ancestral gene order in chloroplast genomes and simulating rearrangements, we have been able to formally test and reject the null hypothesis that *C. reinhardtii *cpDNA has evolved through random rearrangements. Instead, we found that its observed gene order deviates strongly from the degree of sidedness and clustering expected under a random breakage model. *Euglena gracilis *cpDNA also has a high degree of sidedness [33], however, the asymmetry of its coding strand is concentrated in one half of the genome and associated with GC content, which could be influenced by asymmetrical replication of the chromosome [33]. In *C. reinhardtii*, the sidedness is not associated with GC content and we hypothesize that it is driven by co-transcription of genes in a functional cluster. Whereas some clusters of co-transcribed genes (e.g. *rp*l23-*rpl*2-*rps*19, *rpl*16-*rpl*14-*rps*8) were maintained in both *C. reinhardtii *and *C. vulgaris*, novel clusters clearly formed in the *C. reinhardtii *lineage (Figure 4B).

Co-transcription of neighboring genes in the *C. reinhardtii *chloroplast is a widely documented phenomenon. We demonstrated that in addition to the ribosomal protein clusters, global analyses support the elevated level of clustering of other functionally related genes. The aggregate of genes in clusters include most essential genes involved in translation and transcription, and some photosynthetic genes. Coordinated transcription may play a crucial role in the regulation of plastid gene expression in response to light or circadian rhythms [34,35]. It is also possible that some clusters contain *cis*-elements, similar to the artificial polydeoxyadenosine sequences [36], which enhance transcription efficiency. Moreover, most of the putative co-transcription units are not conserved across chlorophytes. Therefore, the majority of functional clusters observed in *C. reinhardtii *represent new gene arrangements.

In the chloroplast gene order phylogeny (Figure 2B), the *C. reinhardtii *lineage resides on a long branch compared to the *C. vulgaris *lineage, and both genomes are more rearranged than that of *N. olivacea*, relative to the common ancestral genome of the three chlorophyte lineages. The elevated rate of chloroplast genome rearrangement in *C. reinhardtii *is associated with invasion of SDRs, which heavily populate the non-coding regions, increasing the total length of the intergenic regions compared to *C. vulgaris *cpDNA by one-third [16]. Although simple sequence repeats are common to microbial genomes [37], such elements are rare in most sequenced chloroplast genomes. Within the *Chlamydomonas *genus (Chlorophyceae), *C. reinhardtii *and *C. gelatinosa *cpDNAs exhibit a prevalence of repetitive DNA and a high degree of gene order variation compared to the *C. moewusii*/*C. pitschmannii *lineage [15,38,39]. The sister lineage to *C. reinhardtii *in our study, *C. vulgaris *(Trebouxiophyceae), contains numerous cpDNA repeat sequences. Besides chlorophyte algae, members of angiosperm families, including Campanulaceae [40], Fabaceae [41,42] and Geraniaceae [43], also contain repeat elements in rearranged cpDNAs, albeit of a much lower copy number [40-43]. These repeat elements may act as molecular "grease" that facilitates non-homologous recombination and creates a pool of diverse genome structures subject to selective retention. Future investigations will test whether the increased rates of rearrangement in plastid genomes with dispersed repeats typically lead to increased sidedness and functional clustering as we infer for *C. reinhardtii*.

Gene order changes reflect relatively rare evolutionary events and are expected to result in much less homoplasy than substitution events in nucleotide or protein sequences over a deep time scale [44]. Phylogeny reconstruction using GRAPPA is highly accurate even for divergent genomes [45], and thus the ancestral gene orders inferred in our study contained sufficient phylogenetic information. The only other software for genome rearrangement phylogeny, BADGER [46], performed poorly on this data set (results not shown). GRAPPA usually inferred unique ancestral gene orders on many data sets we tested. Furthermore, analyses on simulated data have shown that the inferred gene orders scored almost as well as true ancestral gene orders [47]. In our simulation tests of three genomes with 85 genes each, and branch lengths of 50, 20 and 20 (roughly corresponding to the branches leading to *C. reinhardtii*, *C. vulgaris *and *N. olivacea*; see Methods), the average score for ancestral gene orders computed by GRAPPA was only about 7% less than the true scores. In practice, we observed that the less optimal gene orders generally required more rearrangements. Therefore, it is quite likely that any error in our estimation of ancestral gene order has resulted in a downward bias in the inferred number of rearrangements on the branch leading to *C. reinhardtii*. Increasing the number of rearrangements on this branch would only lead to a more certain rejection of the neutrality of rearrangements.

The accuracy of ancestral genome reconstruction also depends on the degree of divergence among extant taxa and taxon sampling. For example, accurate reconstruction of ancestral genomes at the mammalian CFTR locus was achieved at the DNA level [48]. The high-quality reconstruction was attributed to a dense sampling of syntenic genome sequences from eutherian mammals, and the lack of gene order rearrangement at the locus. Because the *C. reinhardtii *cpDNA is one of the most rearranged chloroplast genomes sequenced to date, we included all available chlorophyte chloroplast genomes for evolutionary distance estimation and ancestral gene order reconstruction. The accuracy of our ancestral gene order estimation may improve with inclusion of additional chlorophyte plastid gene orders as they become available, but we do not foresee a substantial reduction in the inferred number of rearrangements separating *C. reinhardtii *and *C. vulgaris *from their common ancestor.

Inversions are thought to be much more common than transpositions in chloroplast genome evolution [27], and our estimation of ancestral genome order was made with the assumption that all rearrangements were inversions. However, we did consider the contribution of inversions and transpositions under different scenarios in the simulation from the ancestral genome. It should be noted that there is not a unique phylogeny distance measure using transposition only, because computationally one transposition is equivalent to two or three inversions [49]. For this reason, we designed our simulations to allow for various ratios of inversion and transposition events. The results of our simulation study did not vary significantly under these scenarios.

The GRAPPA-IR algorithm was developed to account for the inverted repeat (IR) region found in most plastid genomes The IR region seems to evolve at a slower rate in both nucleotide sequence and gene order than the single copy regions [50], and frequent intra-molecular recombination homogenizes the two copies [6,51]. The most conserved gene set in the IR region is the rRNA operon. In IR-containing green plastids, the order of rRNA genes is conserved, but the IR boundaries can vary greatly even within one genus [52]. The IR may restrict rearrangements that cross the boundary of single copy regions, and thus concentrate gene order changes within single copy regions. However, this hypothetical constraint of the IR on genome rearrangements seems to have been lost in the *C. reinhardtii*/*C. vulgaris *lineage. Notably, both lineages have undergone extensive rearrangements since their divergence from a common ancestor, and they contain only a few conserved clusters encoding rRNA or ribosomal proteins. In either genome, genes that typically reside together in the LSC region have often been scrambled and scattered. When comparing the ancestral genome to the *C. vulgaris *gene order, there was no distinction of LSC and SSC regions although many large clusters were still shared (additional file 4). If there were constraints on the breakpoint locations, as experimentally identified in bacterial inversion mutants [53], it would limit the possible paths of evolution, and these constraints on the ancestral gene orders would increase the number of rearrangements relative to the estimations derived from GRAPPA. Therefore, as discussed above, our approach of detecting strong deviation from expectation is conservative in that the number of rearrangements may be underestimated.

Recent studies of plant, animal and fungal genomes have shown that genes involved in the same pathways or genes sharing similar expression patterns are often spatially clustered [1,5,54]. In eukaryotes, the operon structure has only been demonstrated in the nematode *Caenorhabditis *[55]. Comparative analyses of yeast genomes indicate that rearrangements brought together duplicate genes forming the *DAL *cluster involved in allantoin metabolism [56]. In this study, we demonstrated that positive selection for increased clustering has influenced gene order in the chloroplast. Gene clusters, as opposed to separated genes, permit polycistronic transcription and thus fewer transcriptional regulation units. Co-transcription may be facilitated by close spacing of genes in cpDNA because transcription termination is inefficient [57]. Although post-transcriptional RNA processing often creates multiple single-gene transcripts, co-transcription foments an initial stoichiometric accumulation of RNA corresponding to each gene in a cluster. Thus, large clusters can be advantageous in coordinating gene expression on this level.

Experimental approaches are necessary to understand whether these gene clusters function as operons. Because chloroplast primary transcripts are heavily processed – as just one example, the *psb*B cluster in maize accumulates as at least 15 distinct mRNA species with varying translational capacities [58] – direct analysis of the functional advantages of clustering in chloroplasts is challenging. Indeed, *Chlamydomonas *may be a special case, since unlike land plants it has a single rather than multiple RNA polymerases [35]. This situation does not allow differential expression by promoter selectivity, and may therefore serve as a selective force that favors physical grouping of genes rather than evolution of promoter sequences of dispersed genes.

## Conclusion

In conclusion, we infer that gene order in the *C. reinhardtii *plastid evolved in a non-random fashion, and hypothesize that genome structure has been influenced by directional selection acting on variation generated by an increased rate of rearrangement. Our results provide strong evidence that genetic responses to natural selection occur at the level of genome organization. By estimating the ancestral gene order and simulating rearrangements under a null model, we provide a formal demonstration that the chloroplast genome of *C. reinhardtii *has been shaped by natural selection. Although the model of natural selection on gene order remains to be developed, application of our methods to sequences of additional chlorophyte plastid genomes would help to improve the accuracy of the ancestral genome reconstruction and inferred branch lengths. The complex process of gene duplication and loss in bacterial and eukaryotic nuclear genomes presents challenges to reconstruction of ancestral gene orders. Still, the development of new comparative tools [59] gives us hope that the type of analysis presented in this paper will soon be applicable to eukaryotic genomes.

## Methods

### Functional clustering of chloroplast genes

We defined a "functional cluster" as contiguous genes encoded on one strand from one of the following categories: transcription/translation, photosystem I and II, electron transport (cytochrome b6/f complex), and ATP synthase (See additional file 1).

### Kolmogorov-Smirnov test of random clusters

A random cluster consists of genes from any functional category. The n = 85 genes shared in the seven chloroplast genomes shown in Figure 1 were divided into 11 equal sized blocks of *r*_*j *_= 7 genes and one block of 8 genes so that the block sizes and number of blocks are equal. If *m*_*ij *_genes were from the functional category *i *(total *T*_*i *_genes) in the *j*th block, the observed cumulative frequency was *u*_*i *_= ∑_*i*_*m*_*ij*_/*r*_*j*_. The Kolmogorov-Smirnov test measures the deviation of the observed *u*_*i *_from the expected from the random breakage model [13]. The test statistic *D*_*n *_was calculated for each functional category separately.

Dn=max⁡[max⁡(Tin−ui),max⁡(ui−Ti−1n)]
 MathType@MTEF@5@5@+=feaafiart1ev1aaatCvAUfKttLearuWrP9MDH5MBPbIqV92AaeXatLxBI9gBaebbnrfifHhDYfgasaacH8akY=wiFfYdH8Gipec8Eeeu0xXdbba9frFj0=OqFfea0dXdd9vqai=hGuQ8kuc9pgc9s8qqaq=dirpe0xb9q8qiLsFr0=vr0=vr0dc8meaabaqaciaacaGaaeqabaqabeGadaaakeaacqWGebardaWgaaWcbaGaemOBa4gabeaakiabg2da9iGbc2gaTjabcggaHjabcIha4jabcUfaBjGbc2gaTjabcggaHjabcIha4jabcIcaOmaalaaabaGaemivaq1aaSbaaSqaaiabdMgaPbqabaaakeaacqWGUbGBaaGaeyOeI0IaemyDau3aaSbaaSqaaiabdMgaPbqabaGccqGGPaqkcqGGSaalcyGGTbqBcqGGHbqycqGG4baEcqGGOaakcqWG1bqDdaWgaaWcbaGaemyAaKgabeaakiabgkHiTmaalaaabaGaemivaq1aaSbaaSqaaiabdMgaPbqabaGccqGHsislcqaIXaqmaeaacqWGUbGBaaGaeiykaKIaeiyxa0faaa@55C1@

### Phylogeny of chloroplast genomes

Alignments of 50 proteins shared in the 8 chloroplast genomes shown in Figure 2A were concatenated into one data matrix (Additional file 2). 1,000 bootstrap replicates were conducted on the data set using PAUP* 4.0b10 with maximum parsimony and using MEGA with neighbor-joining methods and the Poisson-corrected distance. Maximum likelihood analysis with 100 bootstrap replicates was performed using PHYLIP3.6 with JTT distance and gamma = 0.5. GRAPPA was not used to construct the reference phylogeny.

### Inferring ancestral gene orders

The ancestral gene order was inferred from the gene orders of extant genomes on the best-scored tree following two steps. First, the gene contents for the LSC, SSC and IR regions of ancestral genomes of IR-containing cpDNAs were inferred based on parsimony. Changes in gene copy number due to IR expansion or contraction were considered the last step of gene order changes, and thus the gene contents of ancestral genomes were determined. The ancestral gene orders on the phylogeny for five genomes (excluding *C. vulgaris *and *C. reinhardtii*) were computed using GRAPPA-IR [25], which is a modified version of GRAPPA that scores rearrangements independently within LSC, SSC or IR. Second, the chlorophyte algal gene orders (the extant chloroplast gene orders of *N. olivacea*, *C. reinhardtii *and *C. vulgaris *and the inferred ancestral genome of *N. olivacea *from step one) and the gene order of *M. viride *were used for the inference of the common ancestral gene order of *C. vulgaris *and *C. reinhardtii*. The data set contains duplicated *trn*V-UAC and *trn*G-GCC in *C. vulgaris*, *trn*E-UUC and *psb*A in *C. reinhardtii *and three trans-splicing *psa*A exons in C. *reinhardtii*. The IR regions contained rRNA genes in the same order and orientation in each genome except that one copy was lost in the lineage leading to *C. vulgaris*. To score the genomes with gene duplications and deletions, multiple data sets were created each containing genomes with equalized gene contents by the following assignment rules: one copy of each duplicate genes outside the typical IR was chosen; the IR region lost in *C. vulgaris *was inserted to all possible locations in that genome. Preferably, we should test all these datasets (3,936 total) with inversion medians; however, such computation on one dataset alone will take more than a month. To overcome this limitation, these datasets were computed using breakpoint medians, and the assignment yielded the shortest tree was chosen for a full evaluation by GRAPPA. Because the gene contents of LSC and SSC in *C. reinhardtii *were different from other chloroplast genomes in the study, we allows free rearrangements such that genes in LSC or SSC could commute across the IR.

### Ancestral gene order simulation

A set of simulation experiments were conducted to evaluate the accuracy of ancestral genome reconstruction with long branches. Three genomes with 85 genes each were generated from a defined ancestral gene order, and the branch lengths (inversion distances) were 50, 20 and 20, respectively. The true gene order score was 90 (equals the tree length). The scores were computed for inferred ancestral gene orders by GRAPPA using inversion medians and the random breakage model and then compared to the true score. The experiment was repeated on 30 data sets.

### Random genome rearrangement simulation

Gene orders were simulated under the assumption that the rearrangements involve random breakpoints placed between genes. Initial gene orders were set based on the inferred ancestral gene orders estimated. Random rearrangement operations on the initial genomes were performed for the number of replicates according to the number of rearrangements inferred by GRAPPA. The parameters input to the model were the ratios of inversion and transposition (1:0, 10:1, 1:1) to test the sensitivity of the findings to the specific rearrangement model. The simulated genomes had identical gene content but scrambled gene orders relative to those observed in extant genomes, with the exception that inverted repeats were maintained. Test statistics (below) were calculated for each simulated replicate of 10,000 total and the frequency distributions were used to test the null hypothesis of random rearrangement.

### Sidedness index (C_s_)

We designed the sidedness index (C_s_) to measure the degree to which neighboring genes are clustered on the same strand (side) of the chromosome. A "sided block" includes only adjacent genes on one strand, and the number of sided blocks in a genome is designated as *n*_*SB*_, while the total number of genes is *n*. C_s _is defined as

C_s _= (*n*-*n*_*SB*_)/(*n*-1).

When C_s _reaches the maximum of 1, all genes are located on one side. If every gene resides on the strand opposite its neighbors, C_s _approaches a minimum of zero.

### Functional cluster index (C_f_)

We divided a genome of total *n *genes to *J *sided blocks (*r*_1_, *r*_2_,...*r*_*J*_). In a block, we assigned genes to functional categories. Let the numbers of genes in the *i*th functional category and the *j*th block be *m*_*ij*_, the functional cluster index C_f _is

Cf=1J∑j=1Jrjn∑i=14(mij2)/(rj2).
 MathType@MTEF@5@5@+=feaafiart1ev1aaatCvAUfKttLearuWrP9MDH5MBPbIqV92AaeXatLxBI9gBaebbnrfifHhDYfgasaacH8akY=wiFfYdH8Gipec8Eeeu0xXdbba9frFj0=OqFfea0dXdd9vqai=hGuQ8kuc9pgc9s8qqaq=dirpe0xb9q8qiLsFr0=vr0=vr0dc8meaabaqaciaacaGaaeqabaqabeGadaaakeaacqqGdbWqdaWgaaWcbaGaeeOzaygabeaakiabg2da9maalaaabaGaeGymaedabaGaemOsaOeaamaaqadabaWaaSaaaeaacqWGYbGCdaWgaaWcbaGaemOAaOgabeaaaOqaaiabd6gaUbaadaaeWaqaamaalyaabaWaaeWaaeaafaqabeGabaaabaGaemyBa02aaSbaaSqaaiabdMgaPjabdQgaQbqabaaakeaacqaIYaGmaaaacaGLOaGaayzkaaaabaWaaeWaaeaafaqabeGabaaabaGaemOCai3aaSbaaSqaaiabdQgaQbqabaaakeaacqaIYaGmaaaacaGLOaGaayzkaaaaaiabc6caUaWcbaGaemyAaKMaeyypa0JaeGymaedabaGaeGinaqdaniabggHiLdaaleaacqWGQbGAcqGH9aqpcqaIXaqmaeaacqWGkbGsa0GaeyyeIuoaaaa@50F0@

A larger value of C_f _indicates that functionally related genes are more clustered into blocks.

### RNA analysis

Wild-type CC-124 cells were grown in Tris-Acetate-Phosphate medium [60] under continuous light to mid-log phase. RNA was isolated from 10 mL of cells as previously described [61]. For filter hybridization, 5 μg of total RNA was fractionated in 1.2% agarose and 6% formaldehyde gels, transferred to nylon membranes, and probed with gene-specific PCR products labeled by random priming according to Church and Gilbert [62].

## List of Abbreviations

cpDNA, chloroplast DNA; IR, inverted repeat; SDR, short dispersed repeat

## Authors' contributions

LC conducted the analysis and drafted the manuscript. JLM and CWD conceived the study, helped with the analyses and contributed to the text. LSW contributed the code for the genome simulator. JT carried out the ancestral genome reconstruction. LR conducted the RNA analysis. DBS provided further experimental data review and revision of the draft. All authors read and approved the final manuscript.

## Supplementary Material

Additional File 1 **Gene coding and functional categories**. Text file, lists the names of 85 genes included in the study and corresponding functional categories. Click here for file

Additional File 3 **The gene order data set**. Text file, contains gene orders of seven chloroplast genomes, computed C_s _and C_f _indices, and the inferred rearrangement phylogeny. Click here for file

Additional File 2 **Protein alignment matrix**. Text file, with a NEXUS format data matrix of concatenated proteins from seven chloroplast genomes and the outgroup, *Cyanophora paradoxa*. Click here for file

Additional File 4 **Comparison of gene clusters**. Text file, shows gene clusters shared between the inferred ancestral genome of *C. reinhardtii *and *C. vulgaris *to the cpDNA of *C. vulgaris *and *N. olivacea*. Click here for file

Additional File 5 **Inversions separating *N. tabacum *and *M. polymorpha *cpDNA**. Text file, shows the possible scenarios to transform the chloroplast gene order of *N. tabacum *to *M. polymorpha *cpDNA through inversions. Click here for file
